# Ethical funding for trustworthy AI: proposals to address the responsibilities of funders to ensure that projects adhere to trustworthy AI practice

**DOI:** 10.1007/s43681-021-00069-w

**Published:** 2021-06-13

**Authors:** Allison Gardner, Adam Leon Smith, Adam Steventon, Ellen Coughlan, Marie Oldfield

**Affiliations:** 1 School of Computing and Mathematics, Keele University, Newcastle-under-Lyme, ST5 5BG UK; 2 Dragonfly, London, UK; 3 Health Foundation, London, UK; 4 Artificial Intelligence Group, Royal Statistical Society, London, UK

**Keywords:** Artificial intelligence, Trustworthy, Ethics, Funding, Framework

## Abstract

AI systems that demonstrate significant bias or lower than claimed accuracy, and resulting in individual and societal harms, continue to be reported. Such reports beg the question as to why such systems continue to be funded, developed and deployed despite the many published ethical AI principles. This paper focusses on the funding processes for AI research grants which we have identified as a gap in the current range of ethical AI solutions such as AI procurement guidelines, AI impact assessments and AI audit frameworks. We highlight the responsibilities of funding bodies to ensure investment is channelled towards trustworthy and safe AI systems and provides case studies as to how other ethical funding principles are managed. We offer a first sight of two proposals for funding bodies to consider regarding procedures they can employ. The first proposal is for the inclusion of a Trustworthy AI Statement’ section in the grant application form and offers an example of the associated guidance. The second proposal outlines the wider management requirements of a funding body for the ethical review and monitoring of funded projects to ensure adherence to the proposed ethical strategies in the applicants Trustworthy AI Statement. The anticipated outcome for such proposals being employed would be to create a ‘stop and think’ section during the project planning and application procedure requiring applicants to implement the methods for the ethically aligned design of AI. In essence it asks funders to send the message “if you want the money, then build trustworthy AI!”.

## Introduction

Trustworthy AI^1^ has been a focus in the data science and AI field for several years. It has increased significantly in prominence and urgency with recent controversies involving public sector systems [1] and influencing elections [2].

In the UK, August 2020 was a pivotal month in light of a number of legal cases and decisions challenging the use of some AI and machine learning systems. Examples include the judgement of the UK government visa streaming algorithm in August 2020 and its resultant suspension [3]. This landmark legal challenge highlighted the human rights and equalities that can be caused by some AI systems [4]. Similarly, the ground-breaking case challenging the facial recognition software trialled by South Wales police was upheld on appeal in the first legal case of its kind [5]. Also, in August 2020, we saw the public uproar and legal challenge caused by the algorithm employed to predict grades for students. This clearly indicates that public awareness and impetus to hold AI systems to account is increasing. Around the same time other reports announced the withdrawal of child welfare algorithms by several councils [6] and the suspension of the Most Serious Violence predictive system, part of the £10 million Home Office funded National Data Analytics Solution, by West Midlands Police on the advice of its Ethics Committee [7]. Additionally, many cases have been heard globally and have been upheld [8, 9]. This clearly indicates that public awareness and impetus to hold AI systems to account is increasing.

Despite a general belief that AI systems are more objective and accurate than humans, many algorithms are often not as accurate as claimed, or any more accurate than non-AI systems [10]. They can also be significantly biased leading to discriminatory outcomes, as the cases mentioned above illustrate. Indeed, the language used to describe and sell AI often perpetuates a misleading view of AI quality, a significant concern for systems deemed as ‘high-risk’. High-risk AI applications are those that can potentially result in material harm to an individual or the environment if not correctly deployed e.g. diagnostic AI [11], sentencing [12], recruitment [13], loan approval [14] or chatbots designed to address mental health including addressing suicide [15]. These high-risk AI applications are particularly vulnerable to harms caused by untrustworthy AI. The risks to human rights and indeed life, in the case of medical use of AI [16], increases the urgency to find meaningful mechanisms to change the way we invest in, develop and use AI solutions. If we do not, it is likely that we will continue to see harm occurring and see further litigation.

To illustrate this further, it is worth noting examples of diagnostic and predictive algorithms in the health care setting.

A well-known example is that of a health care risk-prediction algorithm used on more than 200 million US citizens to identify patients who would benefit from “high-risk care management program” [17]. The aim of this management program is to provide chronically ill people specially trained nursing staff and extra primary-care visits. However, this algorithm demonstrated significant racial bias in that Black patients assigned the same level of risk by the algorithm are sicker than a White patient. The authors report that this bias reduced the number of Black patients identified for extra care by more than a half. The reason this bias existed is because one of the features (or rules) the algorithm uses is that of health costs. This feature was used as a proxy for determining health need. However, due to unequal access to healthcare less money is spent on Black patients than equally sick White patients. In effect the algorithm was predicting health care costs not level of illness. The authors also showed that once this issue was noted and the algorithm modified to remove health costs the racial bias was eliminated.

The Covid-19 pandemic has also resulted in many technological solutions and a very recent case has been highlighted with the QCovid living risk prediction algorithm used in the UK. This algorithm estimates risk of hospital admission and mortality from coronavirus 19 in adults [18]. The algorithm used features such as age, ethnicity, deprivation, BMI and a range of comorbidities and had a sensitivity (number of correctly identified positives) of 75.7%. Commendably the authors did test their algorithm performance on men and women (still a rare occurrence) and found little difference. QCovid was used by the Joint Committee on Vaccination and Immunisation (JCVI) to determine who should have priority for the vaccine roll out. However, it has recently been reported [19] by a JCVI committee member that the algorithm was likely to underestimate the risk to vulnerable people suffering from rare disease, particularly younger patients. The result being this group of patients, who are at high risk, were not prioritised for the vaccine. The committee member also pointed out that the datasets used to train the model may have other significant omissions due to some groups effectively shielding and not being exposed to the virus. Although this bias has not been verified it does reveal the importance of transparency and understanding of how such algorithms work if they are to be used to drive healthcare policy.^2^

Indeed, it is not the first algorithm being used to determine vaccine policy to be questioned in this way. Stanford Medicine officials used an algorithm to determine which of their staff should be prioritized for the vaccine. However, it prioritized high-ranking doctors, with little patient-facing contact over residents involved in direct care of Covid-19 patients. The error here was that the junior doctors did not have an assigned location and were young. This resulted in demonstrations by the doctors and public coverage. The leadership stated that they used the algorithm to ensure equity but issued an apology and changed their vaccination policy.

In response to these issues we have seen a significant number of High Level AI Principles (outlined later), frameworks [20] and standards being developed for example IEEE P7010 Transparency of Autonomous Systems [21] from the IEEEE P7000 series [22] and ISO/IEC JTC 1/SC 42 Artificial Intelligence [23]. However, these, in the absence of statutory requirements, are still not enough to prevent the unintentional development of untrustworthy and biased AI systems.

Within the UK we have as yet to meaningfully develop AI specific legislation and regulation. Hence, we still see repeated investment in and use of systems that impact negatively on individuals and groups, despite several government ethics advisory boards and procurement guidelines [24]. This has resulted in a reliance on other regulation such as the GDPR, which focuses on data protection but does address profiling and automated decision making (outlined later). However, the GDPR itself is still struggling with implementation [25] due to the complexity of the guidance and how it specifically addresses the wider issues involving machine learning and AI. A recent analysis of the he UK Information Commissioner Office guidance [26] has concluded that it does not, to date, appear robust enough or sufficiently developed to be deployed meaningfully in the AI space [27].

As well as the lack of regulation forcing the requirements for Trustworthy AI another key issue is the lack of awareness and training in the current field. The main training pipelines and education routes that an AI developer might take do not have benchmark subject statements even as of 2019 [28] which demonstrates a significant gap in addressing data science or AI in Higher Education. There are some areas of good practice, for example the Level 6 Data Science Apprenticeship Standard outlines key knowledge, skills and behaviours addressing ethical development of AI [29]. Professional qualifications are now coming online that also offer future adjustments to professional practice [30]. However, developers need to be aware of, open to and subject to a demand for such qualifications. This therefore leaves the challenge of trying to educate developers and modellers after the fact in a process that, without statutory legislation or professional requirements, is occurring slower than the rate of technological development.

The result of this myriad of issues is the obvious human cost, that we have outlined thus far, plus the significant financial loss to public funds and reputational damage due to the withdrawal of expensive and harmful AI solutions. Therefore, it is vital that we influence ethical development of AI at an as early stage as possible to prevent such problems from occurring.

One way in which we can encourage and ensure the development and deployment of Trustworthy AI systems is to influence public and charitable funding. Significant funding is awarded for AI projects and such grants are hotly sought after. The importance of public funds and investment for AI was addressed in the pivotal Hall-Presenti Review for the DCMS^3^ and BEIS^4^ [31]. The report stressed the importance of public funds used to invest in major challenge areas (identified by Innovate UK and ESPRC) such as personalised and integrated health care. The report also highlighted key issues such as transparency, explainability, training and diversity. Though not expressly mentioned it would be fair to assume that such funding should be driven towards projects that are ethically designed to produce trustworthy AI solutions.

There are many examples of funding calls having ethical requirements (these are outlined later) and additional monitoring, for example value for money is usually taken into account in the funding process. Hence, adjusting this process should not require a significant change in mind-set and requirements for funders than already exists.

Addressing the funding of AI systems may act as a significant nudge to require applicants to educate themselves in and apply Ethical/Trustworthy AI principles and design frameworks. Hence, in response to these issues we propose that grant funding and public tendering of AI systems should require a Trustworthy AI Statement within the grant proposal or tendering document. The statement would outline the actions planned by applicants to ensure their project and/or product can be deemed trustworthy and benchmarked against the rigorous standards.

We acknowledge that this solution is not enough on its own to address the issue of untrustworthy public sector algorithms and that it should be embedded within a wider AI governance and regulatory framework. However, small changes within the operational ecosystem funding for AI will provide the nudge technique that is needed to start to circumvent the problems outlined throughout this paper. This will be a simple, easy to implement, change to the application procedure by funding bodies that could result in a fundamental and vital change to the future of the AI ecosystem.

Essentially the message would be: “if you want the money, then build trustworthy AI”.

## Frameworks for ethical governance of AI

Given that AI and particularly AI ethics is a relatively new field in terms of the wider application of both it may be forgiven that many wishing to utilise or fund AI solutions are not conversant in the potential risks and harms that can occur. However, the AI ethics ecosystem is rapidly developing and providing a large of guidelines, applicable frameworks and tools that researchers can now utilise.

### High level AI principles

Recent years have seen the publishing of a large number of high level AI principles. A report from the Berkman Klein Center for Internet and Society (Harvard University) listed 36 AI principles documents published by organisations such as the United Nations, the OECD, G20, IEEE and EU Commission, though this is certainly not an exhaustive list [32]. Such principles offer normative guidance “for ethical, rights-respecting and socially beneficial AI”. The authors completed a mapping of all 36 principles and 8 central themes:

- Privacy.
- Accountability.
- Safety and security.
- Transparency and explainability.
- Fairness and non-discrimination.
- Human control of technology.
- Professional responsibility.
- Promotion of human values.

One of the examples included by the above report is the Assessment List for Trustworthy Artificial Intelligence (ALTAI) for self-assessment [33]. This was developed by the High-Level Expert Group on Artificial Intelligence (AI HLEG) of the European Commission. The assessment list consists of 7 main categories, each containing subsections as illustrated below^5^ (Table 1):

ALTAI provides a checklist of action points for each section. For example, the Accuracy section consists of 5 checkpoints, two of which are listed below:

- Could a low level of accuracy of the AI system result in critical, adversarial or damaging consequences?
- Did you put in place measures to ensure that the data (including training data) used to develop the AI system is up-to-date, of high quality, complete and representative of the environment the system will be deployed in?

The Data governance section includes questions regarding the production of a data Privacy Impact Assessment, measures to achieve privacy-by-design, data minimisation and:

- Did you align the AI system with relevant standards (e.g. ISO, IEEE) or widely adopted protocols for (daily) data management and governance?

The Explainability section contains the following bullet points:

- Did you explain the decision(s) of the AI system to the users?
- Do you continuously survey the users if they understand the decision(s) of the AI system?

### IEEE ethically aligned design

The IEEE’s Ethically-aligned-design: Prioritizing human wellbeing with autonomous and intelligent systems was created by over 700 global experts [34]. It is noteworthy in that it includes the chapter “Methods to Guide Ethical Research and Design” with subsections on “Interdisciplinary Education and Research”; “Corporate Practices on A/IS” and “Responsibility and Assessment”. The chapter contains a number of recommendations including that ethics training should be a core subject for all those in the STEM field and that relevant accreditation bodies should reinforce an integrated approach to ethics education. The chapter also recommend that:

‘corporations should identify stages in their processes in which ethical considerations, “ethics filters”, are in place before products are further developed and deployed’.

It outlines the example of how ethics review boards would ‘help mitigate the likelihood of creating ethically problematic designs’. The document emphasises the importance of stakeholder involvement in design, the importance of algorithmic transparency and recommend the use of human rights and algorithmic impact assessments. This chapter concludes with the recommendation that full documentation should accompany the final product that addresses auditability, accessibility, meaningfulness and readability and that systems are auditable.

### NHS code of conduct for data-driven health and care technologies

Given that we have looked in more detail at health-related algorithms it is worth noting that the NHS has a code of conduct for data driven technologies [35]. The code consists of 12 sections: (1) how to operate ethically, (2) have a clear value proposition, (3) usability and accessibility, (4) technical assurance, (5) clinical safety, (6) data protection, (7) data transparency, (8) cybersecurity, (9) regulation, (10) interoperability and open standards, (11) generate evidence that the product achieves clinical, social, economic or behavioural benefits and (12) define the commercial strategy.

Principle 1 states that:

‘Increasing use of data-driven technologies, including artificial intelligence could cause unintended harm if we do not think about issues such as transparency, accountability, safety, efficacy, explicability, fairness, equity and bias.’

It references the Data Ethics Framework which informs on the development and adoption of safe, ethical and effective digital and data-driven health and care technologies. It again stresses the over-arching principles of accountability, fairness and transparency and suggests a scoring mechanism for analysing the proposed project. The Data Ethics Framework in turn references the Nuffield Council on Bioethics ‘Ethical principles for data’ highlighting the four principles of:

- Respect for persons.
- Respect for human rights.
- Participation.
- Accounting for decisions.

### The GDPR, AI audits and government guidelines

As stated in the introduction the GDPR has not fully addressed the wider aspects of algorithmic systems, with its understandable focus on data privacy. However, the issues of automated decision making and profiling are mentioned and a number of articles and recitals do have applicability in terms of automated-decisions making (ADM) systems, including profiling. Relevant Articles that reference algorithmic systems directly are Articles 13, 14, 15 and 22. These Articles confer the right to be notified if subject to ADM ‘meaningful information about the logic involved’ including the significance and the envisaged consequences.

Article 22 is focussed on ADM and confers the right to “not be subject to a decision based solely on automated processing, including profiling, which produces legal effects”. As outlined in Article 22 other Articles, which do not explicitly reference ADM also have relevance, such as Article 9 Processing of Special Characteristics. Recital 71 provides further guidance on implementation of Article 22 and references key issues such as fairness, minimisation of errors, transparency and the right to obtain human intervention commonly referred to as human-in-the-loop). Article 35 references the requirement for a Data Privacy Impact Assessments (DPIAs). It has been suggested that the DPIA could be extended to a fuller Algorithmic Impact assessment to allow for a wider assessment of algorithmic risks [36]. Article 35 addresses the importance that the system should be reviewed to assess processing remains in accordance with the DPIA. This is important in considering the ethical design of research projects and their further deployment.

AI Auditing is a developing field that has yet to reach maturity but it is highly likely we will see this become a standard requirement in the future [37]. An AI Audit must be carried out in a rigorous and robust way to ensure fit for purpose models are deployed. The UK’s ICO has published an AI Audit framework [38] that addresses, alongside AI-specific risk areas, the governance and accountability of AI systems including audit trails, training and awareness and compliance. For Humanity have devised the Independent Audit of AI systems, a framework for auditing AI systems (products, services and corporations) by examining the downside risks focussing on Privacy, Bias, Ethics, Trust and Cybersecurity [39].

Data quality is a key aspect of building an effect model, whether AI based or not. The old adage states “rubbish in equals rubbish out”. Data is the bedrock of any model and quality of this data is of paramount concern. The AQuA Book for best practise within the UK Government states “However, if analysis and any supporting models, data and assumptions are not fit for purpose then the consequences can be severe ranging from financial loss through to reputational damage and legal challenge. In the most severe of consequences, lives and livelihoods can be affected.” [40]. In the Lords Enquiry of June 2020 concerns were raised over data availability and quality which went on to impact the models in this area. [55]. The Decision Makers Playbook advises scrutiny of data before any type of decision is made and indeed advises Decision Makers to funny understand their data and seek further advice on the data should they need it [56]. The European Statistical System Committee. (2012). Quality Assurance Framework of the European Statistical System also discusses how to ensure data quality [58] along with the Quality Assurance of Administrative Data (QAAD) framework. These frameworks are not recent and so stand testament to the existing emphasis on data quality.

Guidance and frameworks can also be derived from related areas for example in the UK government guidance on producing quality analysis for government, the AQuA book [40], and governmental recommendations for Business Critical models. It is expected that a model would have a suite of robust supporting paperwork, the burden being higher for business critical models. This guidance was developed post the 2013 MacPherson Review [57] to ensure fit for purpose modelling and could easily be updated and developed for AI, particularly for those in high risk scenarios such as healthcare, along with recommendations from ethics committees and, in particular, the House of Lords Committee on AI. Indeed, as stated earlier, frameworks for best practise data quality also exist and so, if already adhered to, should not provide further unnecessary burden [58]. Legislation for AI systems and mandated regulation, beyond that of data privacy and protection, is also beginning to occur. Most are addressing certain types of AI systems (such as Lethal Autonomous Weapons or self-driving cars) though wider more general AI laws are beginning to be enacted in other countries [41].

### Summary

It is evident that there is a significant body of literature to rely on to enable grant applicants to design proposals incorporating ethics-by-design in the same way as the more established protocols of privacy-by-design [42] and security-by-design [43]. Yet, despite the amount of guidance it is still evident that there is a challenge in putting these frameworks and principles into practise, resulting in the many problems outlined in the introduction.

As there is a growing impetus in the governance and regulation of AI systems, including audits, there will be increasing accountability and indeed, liability for these system. This further increases the urgency to develop a future-proof system of funding and supporting Trustworthy AI projects being developed now to avoid the harms and waste of funds that can occur.

## The role of funders in promoting and ensuring trustworthy AI

There are many examples of AI systems being funded, developed and deployed that are not fit for purpose, unethical, unfair, unsafe and further embedding discrimination in society. A key aspect of ethical AI is that of accountability and often the question is raised as to who should bear ultimate accountability and could potentially be held liable. It may be reasonable to predict that at some point, given the increasing number of challenged AI systems, that questions will be asked of those funding questionable projects. This is particularly relevant to funding using public funds and requirement to adhere to certain public standards.

This is illustrated, for example, by the UK Government Committee on Standards in Public Life report on Artificial Intelligence and Public Standards [44] which stated that:

“Explanations for decisions made by machine learning are important for public accountability. Explainable AI is a realistic and attainable goal for the public sector – so long as public sector organisations and private companies prioritise public standards when they are designing and building AI systems”

And:

“By ensuring that AI is subject to appropriate safeguards and regulations, the public can have confidence that new technologies will be used in a way that upholds the Seven Principles of Public Life^6^”.

Thus, there is a strong moral argument in that it is simply the right thing to ensure the funding of trustworthy AI. As stated earlier grant funding often requires monitoring to ensure value for money and that funded proposals deliver the promised project. Funders therefore have a duty to ensure that AI projects are not untrustworthy thus causing harms and subject to litigation and withdrawal to prevent reputational damage and financial waste.

The UK Parliament Select Committee on Artificial Intelligence has stated in its 2020 report “AI in the UK: No Room for Complacency” [45] that:

“There is a clear consensus that ethical AI is the only sustainable way forward. Now is the time to move the conversation from what are the ethics, to how to instil them in the development and deployment of AI systems”

Hence it is now vital that across the AI lifecycle we need practical operational nudges that will simultaneously educate the AI community^7^ and practically and immediately promote the development of trustworthy AI. One of the most influential ways to achieve this will be to address how AI is funded and how funding bodies manage the process.

In the coming sections we outline practical suggestions that would enable funding bodies to manage the implementation of ethical funding of AI.

### How operational ‘nudges’ can have a wide impact?

One of the solutions we propose in this paper is a simple adjustment to the application procedure which requires a Trustworthy AI Statement, in which applicants must outline their plans to ensure they follow an ethically aligned design approach. Whilst it could reasonably be questioned as to whether such a seemingly small alteration would genuinely cause a difference, there is evidence that small operational changes can have a great effect.

Tackling the lack of diversity in the workplace is a good example to illustrate how smaller ‘nudges’ can have an impact particularly as the lack of diversity within the technology industry is often referred to root cause for why biased systems are developed and deployed before faults are spotted. This is an area despite many decades of initiatives and investment technology still lags significantly behind other sectors.

Given that many of the problems with AI systems can be traced to unconscious bias at all stages of the AI lifecycle it would therefore seem reasonable to suggest that developers undergo unconscious bias or implicit bias training. However, it is now known that unconscious bias training, or implicit bias training does not reduce bias, alter behaviour or change the workplace [46].

Iris Bohnet in her book ‘What works: gender equality by design’ [47] explains that it is easier to change procedures than people and that over time perceptions and opinions will begin to evolve and accept new ways of working. The book highlights the ineffectiveness of unconscious bias training and also how other diversity solutions such as programmes to ‘encourage women’ can actually often just place an extra focus and work burden on women. Such programmes avoid the real structural issues that have resulted in their exclusion in the first place.

One well known example of a small procedural change that did have significant effect is that of “blind” auditions for orchestras. The general consensus pre-1970 was that men were better musicians than women and this explained the lack of representation of women in orchestras (only 5% were women). However, conducting auditions where the interviewers listened behind a screen, thus unaware of the gender of the musician, was effective in redressing this misconception and proved more effective than all unconscious bias training and mentoring in increasing the numbers of female musicians employed (to 35%). So, it is worth considering similar operational and system changes that can be introduced in the funding requirements that could begin to address the problems experienced regarding AI development. No one singular change is enough but each small ‘nudge’ when combined can produce great effect.

## Case studies

Requiring ethical considerations is not an unusual expectation for grant awarding bodies, particularly those within the life science and medical fields. Indeed, any university-led research requiring human involvement requires ethical approval. Below are presented three case studies detailing approaches and methodologies for Ethics Oversight in terms of other aspects such as gender equality, ethics screening and management and formation of specific bioethics boards. The purpose of listing the case studies below is for general consideration of how similar structures could be adapted and applied to AI ethics grant management.

### Case study 1: GCRF and Newton Fund Gender Equality Statement

An example of a simple approach to addressing ethical issues is the Gender Equality Statement required by the £1.5 billion Global Challenges Research Fund (GCRF) and the £735 million Newton Fund managed in the UK by BEIS and with a number of delivery partners including the UKRI and British Council. This fund builds research and innovation partnerships in partner countries to support their economic development and social welfare. As part of the application a Gender Equality Statement is required in which:

“applications must outline how they have taken meaningful yet proportionate consideration as to how the project will contribute to reducing gender inequalities in the Gender Equality Statement section of the application form”.

The statement is allocated a 3500-character section on the application form and should be statement should be project specific, include the projects outputs and outcomes, the make-up of the project team and all other stakeholders, and refer to the processes followed throughout the research process. It cannot be a re-statement of the institution's policy. If the question is considered not applicable, then the statement should explain why. Five criteria are listed in the application guidance:

- Have measures been put in place to ensure equal and meaningful opportunities for people of different genders to be involved throughout the project? This includes the development of the project, the participants in the research and innovation and the beneficiaries of the research and innovation.
- The expected impact of the project (benefits and losses) on people of different genders, both throughout the project and beyond.
- The impact on the relations between people of different genders and people of the same gender. For example, changing roles and responsibilities in households, society, economy, politics, power, etc.
- How any risks and unintended negative consequences on gender equality will be avoided or mitigated against, and monitored.
- Whether any relevant outcomes and outputs are being measured, with data disaggregated by age and gender (where disclosed).

Such weight is given to this section of the application that the application can be rejected if the project proposal is determined to have a negative impact on gender equality or if there is insufficient consideration given within the statement.

As this grant is a broad-based grant it also has a section for applicants to outline the Research Governance and Ethics for the project. This section is subdivided into 3 parts. Firstly, they require an outline of how applicants will ensure the activity will be carried out to the highest standards of ethics and research integrity (2000-characters). Secondly, applicants are requested to outline the potential ethical, health and safety issues (2000-characters). Finally, a sub-section asks if any of the proposed research involves human participation, human tissue, patient/participant data, animal research, genetic and biological risk, arms/military research (including dual-use technologies). If the project does involve any of these aspects, then applicants are required to confirm they have obtained the necessary permission certificates.

### Case study 2: Horizon 2020 ethical checklist

Horizon 2020 is a 79-billion-euro research and innovation fund running from 2014 to 2020 [48]. Its aims are “to ensure that Europe produces world-class science”, “remove barriers to innovation” and “make it easier for public and private sectors to innovate together” to achieve global competitiveness, and to facilitate collaborative innovation so that new projects get off the ground quickly.

Ethics is viewed as an integral part of research from the initial conceptual stage to the finish. As such the Ethics Appraisal Procedure has been used to provide a framework for assessing and conducting an ethically-aligned project, compliant with fundamental ethical principles. It includes an Ethics Review Procedure (which involves ethics screening and assessment) to be conducted before the project start and an Ethics Check and Audit during the implementation phase, summarised in the table below [49] (Table 2).

Applicants are required to complete an Ethics Self-Assessment by completion of an Ethics Issues table^8^ [50]. This is essentially a checklist of actions and list of required documentation. For example in Sect. 4 Protection of Personal Data there is a section addressing profiling:

“- Does it involve profiling, systematic monitoring of individuals or processing of large scale of special categories of data, intrusive methods of data processing (such as, tracking, surveillance, audio and video recording, geolocation tracking etc.) or any other data processing operation that may result in high risk to the rights and freedoms of the research participants?”

Information that is requested for this is:

“1) Details of the methods used for tracking, surveillance or observation of participants.

2) Details of the methods used for profiling.

3) Risk assessment for the data processing activities.

4) How will harm be prevented and the rights of the research participants safeguarded? Explain.

5) Details on the procedures for informing the research participants about profiling, and its possible consequences and the protection measures.”

and documentation requires:

“1) Opinion of the data controller on the need for a data protection impact assessment (art.35 GDPR) (if relevant).”

In the further guidance the checklist states:

“Personal data must be processed in accordance with certain principles and conditions that aim to limit the negative impact on the persons concerned and ensure **fairness**, **transparency** and **accountability** of the data processing, data quality and confidentiality”.

Once the proposal has been submitted and considered for funding the proposal undergoes an Ethics Review. This consists of two phases, an initial Ethics Screening and then, if deemed needed after screening, an Ethics Assessment. This process involves independent ethics experts and qualified staff. The Ethics Review can result in ethics requirements being set as contractual obligations.

As an outcome of the Ethics Review a number of Ethics Requirements and an Ethics Work Package is produced. There are two types of Requirement, those for the grant preparation and then for the ongoing project. The Requirements are included in the grant agreement as project ethics deliverables which are also placed in the work package. If the project breaches the ethics principles an Ethics Audit can occur. Audits can result in changes to the grant agreement and possibly reduction or termination of the grant arrangement.

### Case study 3: MRC Ethics boards

The Medical Research Council (MRC) has a wide range of resources and guidance for researchers in response. One such development, in response to the advances in medicine and biology, is the Nuffield Council on Bioethics, founded in 1991. The purpose of the board is to act as an independent body that will “identify, examine and report on the ethical questions raised by the advances in biological and medical research” [51].

Board funding is currently provided by The Nuffield Foundation, the MRC and the Wellcome Trust. Membership consists of experts from a wide variety of specialisms, including lawyers, educators and philosophers as well as clinical practitioners, leading to a truly multi-disciplinary approach to ethics. The committee provides support with policymaking and addressing public concerns.

In addition, the Ethics, Regulation and Public Involvement Committee (ERPIC) [52] also provides high level ethical oversight and guidance. ERPIC is a council of seven experts who advise on policy relating to a wide range of issues including ethics, legislation and regulation and matters relating to research involving animals or human participation (including personal information).

### Summary

These examples demonstrate that it is possible to insert into an application an additional requirement for applicants to consider a particular ethical aspect. The onus is placed on the applicant to perform the research and find the expertise required to be informed of the specific ethical or governance requirements, such as gender equality issues in case study 1. Case studies 2 and 3 outline a variety of management options where ethical compliance and advice can be provided by a range of qualified staff, experts and ethics boards. Transferring a similar approach to AI ethics would be feasible to do in a fashion similar to the example given.

## Proposals

These proposals were developed in response to the repeated reporting of AI systems that were found to be discriminatory or found to not meet the general claims for the system. The lead author gathered a team of relevant experts to act as co-authors of the proposals. The team includes a researcher with experience of grant-writing and grant review panels, industry standards and AI ethics experts and representatives of a grant funding body.

The proposals primarily address competitive academic funding calls but it should be noted that such calls often are responded to by joint academic, public sector and/or industry teams that have an intended route to impact. Therefore, we recommend that all R&D funding calls relating to AI is subject to a specific trustworthiness assessment. This would take the form of describing how the proposal will address a stated ethical AI framework (either as required by the funding body or chosen by the applicants if not previously specified) and including details of their intended methods. This is outlined by proposal 1 by the insertion of a trustworthy AI statement section in application forms. It should be noted that although there are examples of ethics requirements by funding bodies that cover aspects of AI ethics principles, such as diversity and inclusion and data privacy, none are AI specific and address issues such as explainability of the entire AI system or fairness testing.

It should be stated that these proposals do assume that funders have a degree of accountability in terms of how they choose to direct funds, particularly if this is public money. Additionally, they also have the responsibility to provide the guidance and support to researchers to enable them to respond accordingly. This could be implemented by a simple change to current application forms and guidance (proposal 1), the presence of an AI ethicist on review panels, and/or the constitution of Ethical AI Boards, that could nudge the AI field effectively towards ethical development. Hence, proposal 2 sets out suggestions for funding bodies to consider as to how they would govern this aspect.

Below we outline two key proposal (1) Introduction of a Trustworthy AI Statement and (2) formation of AI Ethics boards.

### Proposal 1: introduction of a trustworthy AI statement

Hundreds of millions of pounds are offered each year to fund AI projects, not including private investment for start-ups. Grant awarding bodies can step in at a fundamental stage of the most innovative aspects of AI development. Requiring applicants to outline the ethical considerations relevant to their project proposal will provide the opportunity for funding bodies, researchers and developers a point of reflection and the opportunity to identify and mitigate potential problems or harms at the outset.

Criteria can be assessed against existing standards such as aqua/GSS standards/European standards as mentioned previously, e.g., the ALTAI self-assessment toolkit and outlined in accompanying guidance for the grant call. It would be expected that sufficient expertise would be present in the review panel to assess the application.

Here we provide a suggested example of such a requirement and the outline guidance that can be made available.

### Sample trustworthy AI statement

Applicants are required to consider the potential negative impacts of their proposed system and to mitigate for potential harms.

Applicants must outline in the Trustworthy AI Statement section of the application form how they have taken meaningful action to ensure the AI project aligns with the principles of ethical and responsible AI design.

The consideration and actions should be specific to the project including justification for the research question; the management of data; the make-up of the project team; the identification and make-up of stakeholders, beneficiaries and groups at risk of bias; the outputs, outcomes and processes to be followed throughout the research programme and plans for deployment. It should not be a re-statement of general policies, though these can be referenced with descriptions as to how the policy will be implemented in the context of the proposed project. Diagrams such as risk matrices with mitigations can be included.

The Trustworthy AI Statement must address the following criteria unless a justification is made why a particular criterion is not applicable.

Criteria:

- Justify why AI is the right approach to the problem they are trying to address
- Have measures in place to ensure equal and meaningful opportunities for people from diverse backgrounds, particularly those known to be under-represented such as women, people of colour and people living with a disability, to be involved throughout the project_._
- The expected impact of the project (benefits and losses) between diverse groups, considering intersectionality; consideration of long term consequences and the approach to managing risks regarding the impact that the technology might have
- *Data set quality control* Consideration and documentation regarding the provenance of data used, any privacy risks and associated mitigations, data diversity and representativeness and data security measures.
- Consideration of algorithmic bias and explanations for metrics and fairness tests use plus details of mitigations for any identified bias and future monitoring and whistle-blower protections.
- Outline how the system will incorporate an Explainable AI approach, avoiding scenarios where the behaviour of a system cannot be explained, after that fact. This is particularly important for projects deemed high impact for example health algorithms. Any use of a system which is not explainable should have a full justification in the context of the rights and freedoms of stakeholders.
- Transparency statement for the project including commitment to disaggregate data and metrics by gender, age and race.
- Clear governance around the development and adoption of the technology

The “insert name of grant award body” reserves the right to reject the application if no Trustworthy AI statement is made or if the proposal is assessed to result in an unfair outcome on different groups.

Resources to aid applicants can be found here “add link to a website/webpage with list of resources/Ethics checklist”

### Proposal 2: management and monitoring by funding bodies

The following are proposals for the management and monitoring of the grants. Many such proposals have parallels in other fields, such as bioethics for example, so operational procedures for instituting these proposals have precedence in some cases:

### Management of grant funding

Each stage of the funding process provides an opportunity to encourage the funding of trustworthy AI. For each funding body, depending on their sector or discipline, different criteria may be necessary to understand the impacts of the proposal. However, funding bodies within sectors may consider adopting a common approach that can be tailored as necessary to facilitate collaboration between funders and reduce the burden for researchers to understand and address the principles of trustworthy AI.

During the funding call:

- Funding bodies support applicants by providing resources, or links to resources that will aid the applicant in considering how to design ethical AI.
- Provide a call specific or general “Ethical AI Checklist” similar to Horizon 2020 Ethics Checklist for project leads to complete, with full cooperation and sponsorship from partner applicants where appropriate.

During selection:

- Review panels should be diverse (as is current practice).
- A review panel should have suitable skills at all stages of the grant approval process to assess the trustworthy statement of application. This is particularly important to provide explanations of reasons for rejection based on inadequate ethical AI statements. This potentially could follow the format of PPIE feedback on panels as it cannot be expected all participants are knowledgeable. However, it is recommended that review panels themselves receive some training or guidance on this aspect of the proposal.

After funds have been awarded:

- Post-funding management of the funded projects should require review of the adherence to the trustworthy statement by grant managers.

### Trustworthy AI boards

- Establish a Trustworthy AI board within (internal) or across funding organisations.
- The board could take on a role in provide resources (as suggested above) and advising on call specific guidance and checklists.
- The board could provide an avenue for safe whistleblowing should any person involved in the project have reservations regarding the nature, risks and harms of the projects going forward.
- Boards could provide expert feedback on proposals during the shortlisting phase with recommendations and feedback or provide representatives to sit on review panels as experts.
- A trustworthy AI board could play a role in supporting fund managers in ongoing monitoring of projects to ensure they do not diverge from original intent and could assess outcomes.
- A high level independent board could provide guidance on policy, arising issues and maintain an overview of the guidance offered by the funding organization. A similar structure exists for example within the MRC with the Ethics, regulation and public involvement committee. Other examples of high level boards could be the Nuffield Council on Bioethics. There are pre-existing organisations that could adapt to a similar role or aid the establishment of such a body. Such a group could liaise with the Standards and regulatory organisations.

## Discussion

The overall approach for proposal 1 is ‘Keep It Simple’ in that it asks for a short section of free text, as opposed to an extensive and complex checklist. This is a core strength as it means that the process is flexible, scalable and, crucially, easy to implement and assess for impact. It aids the applicant in terms of allowing project specific design and inclusion of risk matrices for example and avoids the need for a complicated, integrated approach to ensure that ethical adherence is highlighted in all work streams. Likewise, it also simplifies the review process for the review panel, particularly if specialists are not available. By collating the proposed methods for ensuring the project follows ethical AI principles in one statement it makes it easier for the reviewers to understand and check compliance with their requirements and guidance.

The expectation is that the funding body will provide call specific guidance regarding the expected requirements for ethical design of the proposed AI systems. The funding body may choose to specify an ethical framework that is sector and/or country specific or allow free choice to the applicants to state the framework they intend to work towards. This allows for scaling of the proposal to wider national/international levels. By not specifying a rigid framework we allow for a flexible, adaptable solution that we believe provides a sustainable approach to governance.

Whilst we feel this is a strength of the proposal, and makes the process less onerous for applicants, a limitation may be that funding bodies themselves will require expert support and guidance as to how to design such guidance. This concern led to the development of proposal 2 which includes the utilisation of AI ethics experts as advisors in design of grant calls and on review panels. We also argue that though our proposals maybe seen as a ‘top down’ approach, particular with regards to proposal 2, there is the opportunity to inform and educate in both directions—requiring the grant review bodies and reviewers to understand the process as well as the applicants, who may indeed be the experts. This will therefore influence funders to consider the trustworthy aspects of their calls, to be cognizant of the risks and gather feedback to direct future investment in of AI that has endeavoured to be trustworthy.

Likewise, there is a limitation in terms of applicants who are not fully informed of the principles for ‘ethics-by-design’ and for organisations ethics panels, as is standard in universities and growing in industry, to also fully understand these principles alongside their RRI (responsible research and innovation) frameworks. We recognise that this maybe a difficult hurdle but it is also a core reason why the proposals have been posited. It is hard to educate people who are unaware of the need or value of adhering to such principles. With regards to university ethics boards this is an area that universities will need to consider in their ethical approval and However, it is normal in developing a grant call to collate a team of experts that can either take responsibility of set work packages e.g. ethical governance and stakeholder engagement, in much the same way health calls have nominated PPIE leads. Likewise, organisation ethics boards can also utilise such expert support, as is currently the practise for example with EDI involvement. Hence, we feel this is not an insurmountable obstacle and that by focussing on the funding stream the proposals offer a driver for faster adoption of ethics-by-design approaches outside of any regulatory requirements that maybe forthcoming.

Should a grant funder opt to implement proposal 1 we anticipate that the short-term implication will be an adjustment in the focus of the proposals put forward to meaningfully attempt to address ethics-by design. It will also alter the discussions and grading of grant proposals and potentially direct funding to projects that aim to be ethical and sustainable. This low cost, easy to implement proposal may indeed be as far as the proposals develop, which is a risk. Another concern is that the proposal could become opaque enough that it risks ‘ethics-washing’ proposals. However, we do feel that even this alone will at least enable auditable trails to be created as well as points of reflection and that in the longer-term this could become less of an issue as governance and tools are refined.

Additionally, we appreciate that Proposal 1 may initially cause difficulties for grant applicants such as university researchers and collaborating industry partners who are not au-fait with the wider aspects of developing trustworthy AI. This may inhibit them from applying or indeed re-direct their funding applications to calls that do not have this requirement. However, it is important to note that these proposals would exist within a wider developing infrastructure of AI governance, including the strong likelihood of relevant regulation, as well as sector guidelines and the growth of AI standards, auditing and certification. As it is much harder to retro fit an AI system to adhere to ethical design principles the common sense approach would be to build from concept and design correctly first.

In the medium term we believe, as familiarity and acceptance increases, we would see the practice spread and more funding bodies to develop an established infrastructure of expertise and governance to support researchers. We view that proposal 2 will start to be developed at this point in individual funding organisations or even, if there were to be high level buy in, as a singular governing body akin to bioethics boards. We understand that there is a significant cost implication for the development of proposal 2. However, there are in existence several organisations that can offer support and help design frameworks and formation of governance and advisory boards (e.g. in the UK there is the Ada Lovelace Institute, Turing Institute and the Centre for Data Ethics and Innovation). Hence, in the medium term we feel the impact could be that more calls would enact the proposals and the process begins to become an accepted norm and a backbone of ethical support is provided for both funding bodies and researchers.

We anticipate that in sectors that are highly sensitive to the impact of biased algorithms such as medicine that innovative solutions that provide detailed auditable documentation to outline the trustworthiness and planned governance of the system will have a competitive advantage over others. This will then tie in with the array of procurement guidelines for AI that require ethics adherence and so smooth the transition of ethical innovation from concept, through funding to development, procurement and deployment, with ongoing governance. As such, any resistance will need to adjust to respond to market forces and of course to any legislation coming forward.

In the longer term, whilst we accept this is not a singular cure-all we will see more responsible development and, as the flow of money is affected by these proposals, greater investment directed to the ethical design for trustworthy AI projects. Likewise, as the field develops researchers, developers and funding body representatives will become better educated on the requirements for the development of trustworthy AI.

## Future work

The proposals will be open for review in a stakeholder workshop with participants from funding bodies and academics. Feedback from the stakeholder workshop will be used to modify the proposals and to produce an impact analysis to consider issues raised and proposed mitigations.

Should a funding body choose to introduce the Trustworthy Statement (or a version of it) then it is proposed that the impact should be evaluated. Future research would entail a meta-analysis of the funding of AI projects prior to implementation of the statement and afterwards. This would determine if there were any substantive changes in the types of projects funded, the make-up of the research teams and styles of application. It would also pick out which features of the guidance are enacted, and which do not gain traction. As an adjunct to this it would be pertinent to survey current AI grant award panels for their own knowledge of Trustworthy AI requirements, views of current projects and application procedures and opinion ex-ante of the proposal. Post-hoc, it is proposed that applicants would be surveyed as to their own response to the requirement and how it altered, if at all, attitudes, plans and design of projects. Feedback from the funding organization itself would also be elicited in terms of how the ongoing monitoring of funded projects was managed and any issues therein.

The sharing of quantitative and qualitative themes from successful funding applications can help provide use cases to drive international work to standardise methods, metrics and techniques. We encourage standards bodies and funding bodies to create liaisons to build this feedback loop.

## Conclusion

The purpose of this report is to address the continuing issues that have been seen due to AI being improperly developed and deployed, often leading to harm on individuals and society. We propose that funding bodies incorporate the requirement for trustworthy AI statements in their application procedure. This process will have a two-fold aim of educating funding applicants as to the importance and processes for developing Trustworthy AI systems and hopefully ensuring only those systems that have addressed issues such as bias for example are funded. We have outlined a simple structure and guidance of such a statement that could be modified to suit sector specific requirements.

The overarching outcome would be to have a significant change in the education of researchers in AI Ethics and thus produce research for which risks and harms have been mitigated against. Not only would this prevent harms to individuals, groups and communities caused by poorly developed and biased AI systems but also prevents large amounts of funding to be wasted on projects that eventually need to be withdrawn.

**Table 1:**
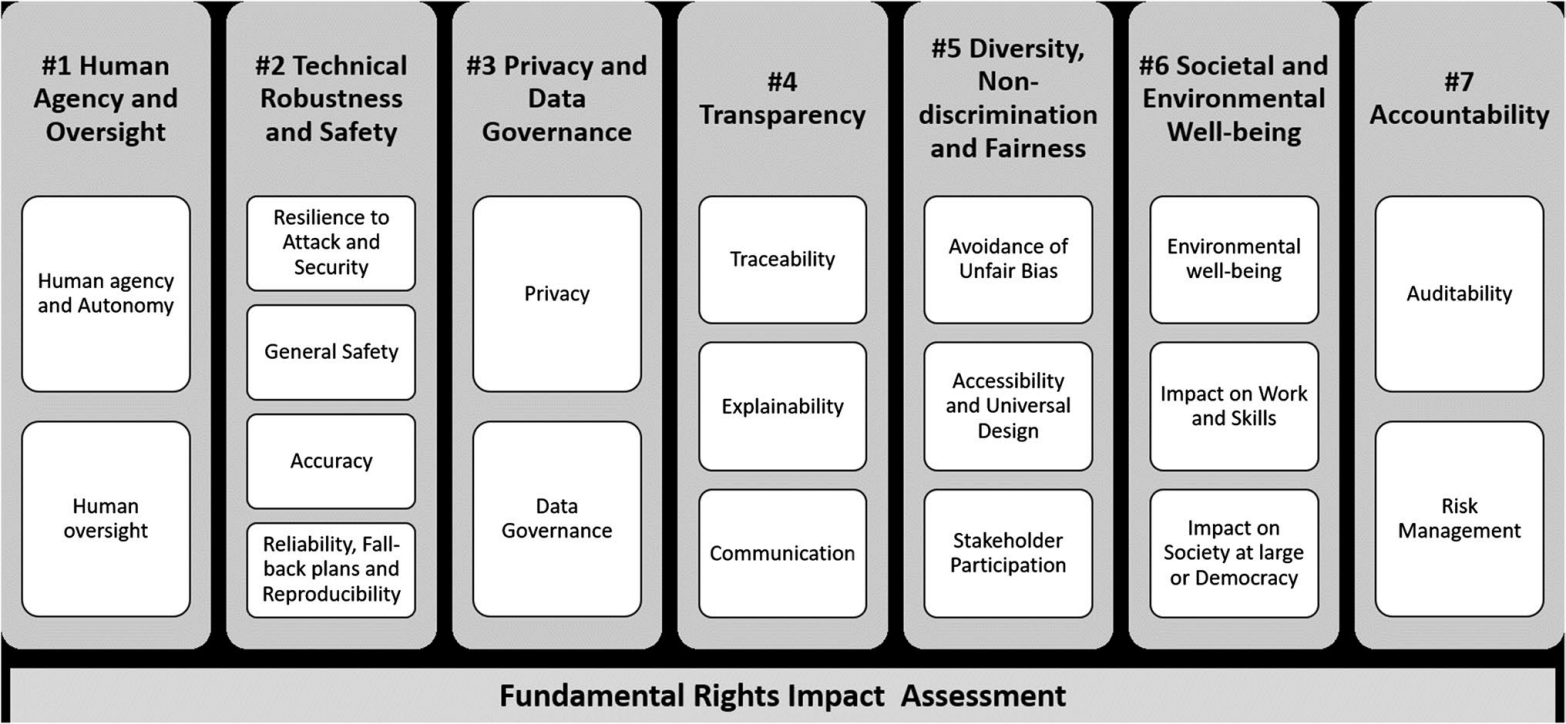
Summary of categories in Assessment List for the Trustworthy Artificial Intelligence (ALTAI) for self-assessment

**Table 2:** Summary of ethics appraisal steps for the horizon 2020 grant fund

Activity	Who?	When?	How?
Ethics self-assessment	Applicant	Application phase	Consideration of ethical issues of the proposal
Ethics pre-screening/screening	Ethics experts and/or qualified staff	Evaluation phase	Review of application material
Ethics assessment (for proposals involving hESC or raising serious ethical issues: severe intervention on humans)	Ethics experts	Evaluation/grant preparation phase	Review of application material
Ethics check/audit	Ethics experts	Implementation phase	Review of project deliverables/interview with applicants

## Footnotes

1 The definition for Artificial Intelligence (AI) is open to debate, with the definition shifting over time as new advancements occur. For the purpose of this paper we follow the EU Commission HLEG in AI definition “Artificial intelligence (AI) refers to systems that display intelligent behaviour by analysing their environment and taking actions – with some degree of autonomy – to achieve specific goals. AI-based systems can be purely software-based, acting in the virtual world (e.g. voice assistants, image analysis software, search engines, speech and face recognition systems) or AI can be embedded in hardware devices (e.g. advanced robots, autonomous cars, drones or Internet of Things applications).” [53]. We include Machine Learning (ML) in this definition. We define ML as the use of computer algorithms to automatically analyse and learn from large datasets to achieve specific outcomes such as predictions and decisions.

2 The Ada Lovelace Institute have recommended that the government engage experts to form the Group of Advisors on Technology in Emergencies (GATE), an advisory body that would act in a similar way to SAGE [54].

3 Department for Digital, Culture, Media and Sport.

4 Department for Business, Energy and Industrial Strategy.

5 A point to note with this framework is that it requires a Human Rights Impact Assessment at the outset. This is significant as many of the legal actions being levied against some AI systems are doing so under human rights, privacy or equalities legislation.

6 Seven Principles of Public Life: honesty, integrity, openness, leadership, selflessness and accountability.

7 The AI Community refers to all those involved in conceiving, funding, procuring, developing, deploying and using the system.

8 Not all sections of the checklist are relevant for all projects, with some sections addressing embryo and animal research for example.

## References

[1] Clement-Jones, I.: The government’s approach to algorithmic decision-making is broken: here’s how to fix it. The Guardian (2020). https://www.theguardian.com/technology/2018/mar/17/facebook-cambridge-analytica-kogan-data-algor

[2] Cadwallader, C.: How Cambridge Analytica turned Facebook ‘likes’ into a lucrative political tool. The Guardian (2018). https://www.theguardian.com/technology/2018/mar/17/facebook-cambridge-analytica-kogan-data-algorithm

[3] Anon: Home Office drops ‘racist’ algorithm from visa decisions. BBC (2020). https://www.bbc.co.uk/news/technology-53650758

[4] Anon: We won! Home office to stop using racist visa algorithm. Joint Council for the Welfare of Refugees (2020). https://www.jcwi.org.uk/news/we-won-home-office-to-stop-using-racist-visa-algorithm

[5] Liberty: Liberty wins ground-breaking victory against facial recognition tech. Liberty [Online] (2020). https://www.libertyhumanrights.org.uk/issue/liberty-wins-ground-breaking-victory-against-facial-recognition-tech/

[6] Redden J (2020). Datafied child welfare services: unpacking politics, economics and power. Policy Studies.

[7] West Midlands Police and Crime Commissioner: Ethics Committee Meeting September 2020. West Midlands Police Commissioner (2020). https://www.westmidlands-pcc.gov.uk/archive/ethics-committee-meeting-september-2020/

[8] Henry, D.: Electrician fired for refusing to use facial scanning system wins $23000. NZ Herald (2019). https://www.nzherald.co.nz/business/electrician-fired-for-refusing-to-use-facial-scanning-system-wins-23000/VCVCND6KZH5JDSEIOBQLOX4B7A/

[9] Peters, J.: IBM will no longer offer, develop, or research facial recognition technology. The Verge (2020). https://www.theverge.com/2020/6/8/21284683/ibm-no-longer-general-purpose-facial-recognition-analysis-software

[10] Nagendran, M., et al.: Artificial intelligence versus clinicians: systemic review of design, reporting standards, and claims of deep learning studies. BMJ **368**, m689 (2020) 10.1136/bmj.m689 PMC7190037 32213531

[11] Liu X, Faes L, Kale A, Wagner S, Fu D (2019). A comparison of deep learning performance against health-care professionals in detecting diseases from medical imaging: a systematic review and meta-analysis. Lancet Digital Health.

[12] Deeks, A.: The judicial demand for explainable artificial intelligence. Columbia Law Rev. 1829–1850 (2019)

[13] Holmes, A.: AI could be the key to ending discrimination in hiring, but experts warn it can be just as biased as humans. Bus. Insider (2019). https://www.businessinsider.com/ai-hiring-tools-biased-as-humans-experts-warn-2019-10?r=US&IR=T

[14] Turiel, J.D., Aste, T.: Peer-to-peer loan acceptance and default prediction with artificial intelligence. Royal Society Publishing (2020). https://royalsocietypublishing.org/. 10.1098/rsos.191649 10.1098/rsos.191649 PMC7353984 32742678

[15] Robinson, J., Thorn, P.: Do chatbots have a role to play in suicide prevention? The conversation (2018). https://theconversation.com/do-chatbots-have-a-role-to-play-in-suicide-prevention-105291

[16] Knoppers B, Thorogood A (2017). Ethics and big data in health. Curr. Opin. Syst. Biol..

[17] Obermeyer Z, Powers B, Vogeli C, Mullainathan S (2019). Dissecting racial bias in an algorithm used to manage the health of populations. Science.

[18] Clift, A., Coupland, C.E.A.: Living risk prediction algorithm (QCOVID) for risk of hospital admission and mortality from coronavirus 19 in adults: national derivation and validation cohort study. BMJ **371**, m3731 (2020) 10.1136/bmj.m3731 PMC7574532 33082154

[19] Meaker, M.: Algorithm used to set vaccine priority order missed key vulnerable groups. The Telegraph (2020). https://www.telegraph.co.uk/technology/2021/01/10/algorithm-used-set-vaccine-priority-order-missed-key-vulnerable/

[20] Anon: Framework to Review Models. National Audit Office (2016). https://www.nao.org.uk/wp-content/uploads/2016/03/11018-002-Framework-to-review-models_External_4DP.pdf. Accessed 11 Mar 2016

[21] IEEE P7001 Working Group: IEEE P7001 transparency of autonomous systems. IEEESA (2021). https://sagroups.ieee.org/7001/ Accessed 20 Jan 2021

[22] IEEE SA: Ethics in action in autonomous and intelligent systems. IEEE (2020). https://ethicsinaction.ieee.org/p7000/

[23] ISO/IEC: ISO/IEC JTC 1/SC 42 Artificial intelligence. BSI (2021). https://www.iso.org/committee/6794475.html

[24] Gardner, A.: Don’t write off government algorithms—responsible AI can produce real benefits. The Conversation (2020). https://theconversation.com/dont-write-off-government-algorithms-responsible-ai-can-produce-real-benefits-145895

[25] Mikkelson, D., Soller, D.H., Strandell-Jansso, M., et al.: Companies must automate and streamline, or the challenge of GDPR compliance will overwhelm them. McKinsey (2019). https://www.mckinsey.com/business-functions/risk/our-insights/gdpr-compliance-after-may-2018-a-continuing-challenge

[26] Anon: Explaining decisions made with AI. Information Commisioners Office (2020). https://ico.org.uk/for-organisations/guide-to-dp/key-data-protection-themes/explaining-decisions-made-with-artificial-intelligence/. Accessed 10 Jan 2021

[27] Kazim, E., Koshiyama, A.: Explaining decisions made with AI: a review of the co-badged guidance by the ICO and the Turing Institute. SSRN (2020). https://papers.ssrn.com/sol3/papers.cfm?abstract_id=3656269. Accessed 26 Aug 2020

[28] QAA: Subject benchmark statement: Computing. QAA (2019). https://www.qaa.ac.uk/docs/qaa/subject-benchmark-statements/subject-benchmark-statement-computing.pdf?sfvrsn=ef2c881_10. Accessed Oct 2019

[29] Anon: Data scientist (Integrated Degree). Institute for Apprenticeship and Technical Education (2019). https://www.instituteforapprenticeships.org/apprenticeship-standards/data-scientist-(integrated-degree)-v1-0. Accessed 10 Jul 2019

[30] CERTNEXUS: Certified Ethical Emerging Technologist Professional Certificate. Coursera (2021). https://www.coursera.org/professional-certificates/certified-ethical-emerging-technologist. Accessed 3 Jan 2021

[31] Hall, H., Presenti, J.: Growing the artifical intelligence industry in the UK. UK Government (2017). https://www.gov.uk/government/publications/growing-the-artificial-intelligence-industry-in-the-uk. Accessed 15 Oct 2017

[32] Fjeld, J., Nagy, A.: Principled artificial intelligence. Harvard Publishing (2020). https://cyber.harvard.edu/publication/2020/principled-ai. Accessed 15 Jan 2020

[33] AI HLEG: Assessment list for trustworthy artificial intelligence (ALTAI) for self-assessment European Commission (2020). https://ec.europa.eu/digital-single-market/en/news/assessment-list-trustworthy-artificial-intelligence-altai-self-assessment (2020)

[34] IEEE Standards Association: Ethically Aligned Design Version 2. IEEE (2019). https://standards.ieee.org/industry-connections/ec/ead-v1.html

[35] Department of Health and Social Care: Code of conduct for data driven health and care technology. UK Government (2020). https://www.gov.uk/government/publications/code-of-conduct-for-data-driven-health-and-care-technology/initial-code-of-conduct-for-data-driven-health-and-care-technology

[36] Kaminski, M.E., Gianclaudio, M.: Algorithmic impact assessments under the GDPR: producing multi-layered explanations. Int. Data Privacy Law **ipaa020**, 6 (2020)

[37] Morley J (2020). From What to how: an initial review of publicly available AI ethics tools, methods and research to translate principles into practices. Sci. Eng. Ethics.

[38] Binns, R.: An overview of the auditing framework for artificial intelligence and its core components. Information Commissioner Office (2019). https://ico.org.uk/about-the-ico/news-and-events/ai-blog-an-overview-of-the-auditing-framework-for-artificial-intelligence-and-its-core-components/. Accessed 26 Mar 2019

[39] Carrier, R.: Independent audit of AI systems—FAQ. ForHumanity (2019). https://www.forhumanity.center/blog-posts/2019/8/8/independent-audit-of-ai-systems-faq. Accessed 8 Aug 2019

[40] HM Treasury: The Aqua Book: guidance on producing quality analysis for government. UK Government (2015). https://www.gov.uk/government/publications/the-aqua-book-guidance-on-producing-quality-analysis-for-government. Accessed 26 Mar 2015

[41] Walch, K.: AI laws are coming. Forbes (2020). https://www.forbes.com/sites/cognitiveworld/2020/02/20/ai-laws-are-coming/?sh=34910dda2b48. Accessed 20 Feb 2020

[42] van Rest, J., et al.: Designing privacy by design. privacy technologies and policy. Lect. Notes Comput. Sci. **8319**, 55–72 (2014)

[43] Chehrazi, G., Heimbach, I., Hinz, O.: The impact of security by design on the success of open source software. Research Papers, vol. 179 (2016)

[44] The Committee of Standards in Public Life: Artificial Intelligence and Public Standards: report. Uk Parliament (2020). https://www.gov.uk/government/publications/artificial-intelligence-and-public-standards-report. Accessed 10 Feb 2020

[45] House of Lords Liaison Committee: AI in the UK: No room for complacency. UK Parliament (2020). https://publications.parliament.uk/pa/ld5801/ldselect/ldliaison/196/19602.htm. Accessed 18 Dec 2020

[46] Dobbin, F., Kalev, A.: Why doesn’t diversity training work? Anthropol. Now **10**, 48–55 (2018)

[47] Bohnet, I.: What works: gender equality by design. Belknap Press of Harvard University Press, Cambridge (2016)

[48] Innovate UK and UK Research and Innovation: Horizon 2020: what it is and how to apply for funding. UK Government (2020). https://www.gov.uk/guidance/horizon-2020-what-it-is-and-how-to-apply-for-funding. Accessed 24 Dec 2020

[49] European Commission: Ethics: European Commission (2020). https://ec.europa.eu/research/participants/docs/h2020-funding-guide/cross-cutting-issues/ethics_en.htm. Accessed 14 Jan 2021

[50] Directorate-General for Research and Innovation: Horizon 2020 Programme: How to complete your ethics self-assessment. European Commission (2020). https://ec.europa.eu/research/participants/data/ref/h2020/grants_manual/hi/ethics/h2020_hi_ethics-self-assess_en.pdf. Accessed 4 Feb 2019

[51] Medical Research Council: The Nuffield council on bioethics. UKRI (2020). https://mrc.ukri.org/research/policies-and-guidance-for-researchers/the-nuffield-council-on-bioethics/

[52] Medical Research Council. Ethics, Regulation & Public Involvemnet Committee (ERPIC). UKRI (2020). https://mrc.ukri.org/research/policies-and-guidance-for-researchers/erpic/#:~:text=The%20Ethics%2C%20Regulation%20and%20Public,issues%20relating%20to%20medical%20research

[53] EU Commission: A definition of artificial intelligence: main capabilities and scientific disciplines. Eu Commission (2019) https://ec.europa.eu/digital-single-market/en/news/definition-artificial-intelligence-main-capabilities-and-scientific-disciplines. Accessed 9 Apr 2019

[54] Ada Lovelace Institute: COVID-19 rapid evidence review: Exit through the App Store. Ada Lovelace Institute (2020). https://www.adalovelaceinstitute.org/evidence-review/covid-19-rapid-evidence-review-exit-through-the-app-store/. Accessed 19 Apr 2020

[55] Lords Select Committee on Science and Technology: Lords select committee on science and technology afternoon session—co-rected oral evidence: The Science of COVID-19 (London, 2 June 2020) (2020). https://committees.parliament.uk/oralevidence/444/pdf/

[56] The Decision Makers Playbook Publisher: Pearson Education Limited (2020) (ISBN: 9781292129334)

[57] HM Treasury: Review of quality assurance of government analytical models. (2013). https://assets.publishing.service.gov.uk/government/uploads/system/uploads/attachment_data/file/206946/review_of_qa_of_govt_analytical_models_final_report_040313.pdf

[58] European Statistical System Committee: Quality assurance framework of the european statistical system (2012)

